# EBV and MSI Status in Gastric Cancer: Does It Matter?

**DOI:** 10.3390/cancers15010074

**Published:** 2022-12-22

**Authors:** Catarina Neto do Nascimento, Luís Mascarenhas-Lemos, João Ricardo Silva, Diogo Sousa Marques, Catarina Ferreira Gouveia, Ana Faria, Sónia Velho, Rita Garrido, Rui Maio, Andreia Costa, Patrícia Pontes, Xiaogang Wen, Irene Gullo, Marília Cravo, Fátima Carneiro

**Affiliations:** 1 Department of Gastroenterology, Hospital Beatriz Ângelo, 2674-514 Loures, Portugal; 2 Faculty of Medicine, Catholic University of Portugal, 2635-631 Rio de Mouro, Portugal; 3 NOVA Medical School, Universidade NOVA de Lisboa, 1169-056 Lisboa, Portugal; 4 Department of Pathology, Hospital da Luz de Lisboa, 1500-650 Lisboa, Portugal; 5 Faculty of Medicine, University of Porto (FMUP), 4200-319 Porto, Portugal; 6 Department of Oncology, Hospital Beatriz Ângelo, 2674-514 Loures, Portugal; 7 Department of Dietetics and Nutrition, Hospital Beatriz Ângelo, 2674-514 Loures, Portugal; 8 Department of General Surgery, Hospital Beatriz Ângelo, 2674-514 Loures, Portugal; 9 Department of General Surgery, Hospital da Luz de Lisboa, 1500-650 Lisboa, Portugal; 10 Department of Oncology, Centro Hospitalar Universitário de São João (CHUSJ), 4200-319 Porto, Portugal; 11 Department of Pathology, Centro Hospitalar Universitário de São João (CHUSJ), 4200-319 Porto, Portugal; 12 i3S—Instituto de Investigação e Inovação em Saúde and Institute of Molecular Pathology and Immunology, University of Porto (Ipatimup), 4200-135 Porto, Portugal; 13 Department of Pathology, Centro Hospitalar de Santo António, 4099-001 Porto, Portugal; 14 Department of Gastroenterology, Hospital da Luz de Lisboa, 1500-650 Lisboa, Portugal; 15 Faculty of Medicine, University of Lisbon (FMUL), 1649-028 Lisboa, Portugal

**Keywords:** gastric cancer, Epstein–Barr virus, microsatellite instability, neoadjuvant chemotherapy, perioperative chemotherapy predictor, prognosis, molecular subtype, gender, females

## Abstract

**Simple Summary:**

Gastric cancer is characterized by high morphologic and molecular heterogeneity. Microsatellite instability (MSI-high) and Epstein–Barr-virus-positive (EBV+) tumors have been associated with better prognosis, but the benefit of perioperative chemotherapy (POPChT) in these tumors is still debatable. Moreover, recent evidence has suggested that response to treatment and prognosis are sex-modulated. We aimed to evaluate the prognostic and predictive value of tumor-specific molecular subtypes on survival and response to POPChT. In our cohort, we did not find differences in overall survival and progression-freesurvival between microsatellite stable (MSS)/EBV-, MSI-high or EBV+ tumors in patients submitted to direct surgery or POPChT. However, females with MSI-high tumors showed a significantly better OS than females with MSS tumors when submitted to POPChT, while in males, the opposite behavior was observed. Thus, our findings may help to pave the way to personalized treatment in GC, considering both patients’ and disease characteristics.

**Abstract:**

We investigated the impactof microsatellite instability (MSI) and Epstein–Barr virus (EBV) status in gastric cancer (GC), regarding response to perioperative chemotherapy (POPChT), overall survival (OS), and progression-free survival (PFS). We included 137 cases of operated GC, 51 of which were submitted to POPChT. MSI status was determined by multiplex PCR and EBV status by EBV-encoded RNA in situ hybridization. Thirty-seven (27%) cases presented as MSI-high, and seven (5.1%) were EBV+. Concerning tumor regression after POPChT, no differences were observed between the molecular subtypes, but females were more likely to respond (*p* = 0.062). No significant differences were found in OS or PFS between different subtypes. In multivariate analysis, age (HR 1.02, IC 95% 1.002–1.056, *p* = 0.033) and positive lymph nodes (HR 1.82, IC 95% 1.034–3.211, *p* = 0.038) were the only prognostic factors for OS. However, females with MSI-high tumors treated with POPChT demonstrated a significantly increased OS compared to females with MSS tumors (*p* = 0.031). In conclusion, we found a high proportion of MSI-high cases. MSI and EBV status did not influence OS or PFS either in patients submitted to POPChT or surgery alone. However, superior survival of females with MSI-high tumors suggests that sex disparities and molecular classification may influence treatment options in GC.

## 1. Introduction

Gastric cancer (GC) is the fifth most common cause of cancer-related death worldwide, with Portugal being one of the Western European countries with the highest incidence and mortality [1]. Most GCs are diagnosed at advanced stages, which accounts for a dismal prognosis [2,3]. GC is characterized by genetic, morphologic, and molecular heterogeneity [4,5,6], which may influence therapeutic decisions. 

From the morphological standpoint, the two most used classifications in clinical practice are the Laurén and the World Health Organization (WHO) classifications [7,8]. However, molecular characterization of GC has been gaining momentum, andthe identification of new clinical biomarkers and therapeutic targets is becoming fundamental. The Cancer Genome Atlas (TCGA) research network identified four molecularly distinct GC subtypes: Epstein–Barr virus (EBV)-positive (EBV+), microsatellite instability-high (MSI-high),genomically stable (GS), and tumors with chromosomal instability (CIN) [5]. Other genomic classifications, such as the Asian Cancer Research Group subtyping, show some overlap with the TCGA classification [9]. 

MSI-high GCs are characterized by DNA hypermethylation and *MLH1* silencing [4,5,10,11]. The frequency of MSI-high GC reported in the literature has a considerable variation, ranging between 7% and 50%. Putative reasons for this variability include epidemiologic differences of enrolled patients in distinct cohorts (e.g., Asian versus Caucasian), inconstant tumor stage distribution between clinical trials, and differences in the methods used for investigating MSI or mismatch repair protein (MMR) status [5,12,13,14,15,16,17,18]. However, recent studies from Europe and clinical trials have reported a lower percentage of MSI-high cases (6.6–11.7%) [12,13,19,20,21,22,23], compared to the TCGA study in which MSI-high tumors accounted for 22% of cases [5]. Concerning tumor stage, MSI-high tumors are associated with non-metastatic and negative nodal disease [9,11,17]. They are more prevalent in older female patients and usually located in the gastric antrum [5,9,24]. Morphologically, MSI-high tumors have been associated with intestinal (tubulo/papillary) histological subtype, solid (poorly differentiated) morphology, as well as the presence of prominent lymphoid infiltrate [8,25,26].

EBV+ GC displays extreme DNA hypermethylation in the EBV-CpG island methylator phenotype (EBV-CIMP); recurrent *PIK3CA* mutations; amplification of *JAK2*, *CD274* (also known as PD-L1) and *PDCD1LG2* (also known as PD-L2); *CDKN2A* silencing and altered immune cell signaling [5]. EBV+ tumors represent approximately 7–9% of GCs, are more prevalent in males, and more often have a proximal location [4,5,6,15,22,27]. They frequently display features of GC with lymphoid stroma [28,29] and are strongly associated with PD-L1 positivity, with potential therapeutic implications [28,30]. 

Strategies and therapeutic options to improve survival in GC have evolved, and in Europe, perioperative chemotherapy (POPChT) (neoadjuvant and adjuvant) with a fluoropyrimidine-oxaloplatin and taxane (FLOT) regimen is currently recommended for patients with stage ≥IB resectable GC [3,31,32]. Other strategies are used to improve survival, mainly adjuvant chemotherapy (ChT) in Asia and adjuvant chemoradiation in North America [31]. At this time, treatment approaches in resectable GC are based solely on clinical TNM staging, while patients’ characteristics and tumor subtypes are not accounted for. Identification of biomarkers to guide our decisions and selection of perioperative treatment is an unmet need.

Although MSI-high and EBV+ GCs have been associated in most studies with improved survival, as compared to those with MSS/EBV−, evidence for these molecular subtypes on prognosis is still conflicting [5,19,20,23,33,34,35,36]. Recent evidence has suggested that the favorable prognostic value of MSI-high tumors might be only observed in female patients [37,38]. 

The benefit of POPChT in MSI-high GC is even more controversial. Data from post hoc analysis of phase III MAGIC trial, published by Smyth EC et al. in 2017, showed that MSI-high tumors had a positive prognostic effect in patients treated with surgery alone but a worse survival outcome when treated with surgery plus ChT [19]. However, these conclusions were based on the analysis of 20 MSI-high cases, only 9 submitted to neoadjuvant ChT and only 4 of these having completed adjuvant ChT. Several studies followed examining these worrisome findings, but a number of issues should be taken into consideration before assuming definitive conclusions: (1) most studies published after the study by Smith et al. were performed in Asia, where GC is diagnosed usually at early stages; (2) surgical lymphadenectomies differ betweenEuropean and North American Centers; (3) in North America, patients are usually treated with adjuvant chemoradiation instead of perioperative regimens [34,39]. 

Probably due to these controversies, ESMO 2022 guidelines do not exclude patients with MSI-high tumors from neoadjuvant ChT in the setting of POPChT with FLOT regimen [3].

The purpose of this study is to further explore clinical implications of the TCGA molecular classification, focusing on EBV+ and MSI-high molecular subtypes, the identification of which is accurately reproducible in clinicopathological practice. Our specific objectives were to assess the clinicopathological characteristics of EBV+ and MSI-high subtypes, as compared to MSS/EBV−, regarding prognosis and response to neoadjuvant ChT. 

## 2. Materials and Methods

### 2.1. Study Design and Data Collection

A total of 137 cases of GC were retrospectively selected from two Portuguese hospitals: Hospital Beatriz Ângelo (HBA) and Centro Hospitalar Universitário de São João (CHUSJ). Exclusion criteria encompassed carcinomas of the gastroesophageal junction and gastric stump. Formalin-fixed and paraffin-embedded (FFPE) blocks of tumor samples from surgical specimens (with or without neoadjuvant ChT) were obtained, between 2004 and 2018. 

Data were retrospectively collected from the medical charts. For each patient, we recorded demographic data, clinical staging, neoadjuvant ChT protocols, type of surgery, adjuvant ChT or chemoradiotherapy, progression status, and survival. 

Pathological data were collected from the available reports; FFPE blocks were retrieved, and all slides were reviewed by two pathologists with experience in gastric pathology. The recorded aspects included tumor location, tumor size, histological classification (according to WHO 2019 and Laurén’s classifications), perineural invasion (PNI), lymphovascular invasion (LVI), total number of lymph nodes (LN), number of lymph nodes with metastases, pathological staging, surgical resection margins status (R), and if applicable, histologic tumor regression grade (TRG) after neoadjuvant ChT (Becker’s grading system) [40]. Pathological tumor–nodes–metastasis (pTNM) categories and staging were classified according to the eighth edition American Joint Committee on Cancer (AJCC) system for GC [41].

### 2.2. EBER In Situ Hybridization for Epstein–Barr Virus

Epstein–Barr encoding region (EBER) in situ hybridization was the methodology of choice for the detection of the Epstein–Barr virus (EBV) in tissue sections. Positive (EBV-infected) cases showed diffuse nuclear staining of cancer cells. The technique was performed with the fully automated Ventana™ BenchMark ULTRA System (Ventana Medical Systems, Tucson, AZ, USA) using the clone Eber 1 Dnp Probe (Roche-Mannheim, Germany).

### 2.3. MSI Status

The surgical specimen samples were all processed at Ipatimup Diagnostics Laboratory for PCR-base molecular analysis, which is considered the gold standard for MSI status evaluation [42]. Hematoxylin and eosin-stained slides cut from formalin-fixed, paraffin-embedded tissues were evaluated by a pathologist for tumor cell content. The tumor representative area was identified and macro dissected using a scalpel. DNA was extracted using Promega’s Maxwell FFPE system, as recommended by the manufacturer. After this step, each DNA sample was quantified using a Nanodrop 2000 Spectrophotometer, and according to the DNA concentration, between 10 and 50 ng of DNA was used for the PCR reaction. For MSI analysis, a panel of five monomorphic markers (*BAT-25*, *BAT-26*, *NR-21*, *NR-24*,and *NR-27*) was used [43]. Multiplex PCR was performed using Quiagen’s Master Mix enzyme. Fragments were then analyzed through capillary electrophoresis using a 3130XL Sequencer (Life Technologies, California, CA, USA) and analyzed in the respective software. For interpretation purposes, microsatellite instability at ≥2 loci was defined as MSI-high, instability at a single locus was defined as MSI-low, and if there was no instability at any of the loci, the case was defined as MSS. MSI-low cases were grouped with MSS cases for further analysis, according to the revised Bethesda Guidelines [42]. 

### 2.4. Statistical Analysis

Chi-squared tests or Fisher’s exact tests were used to compare categorical variables. For multiple categorical variables, the Bonferroni multiple comparisons correction was applied to the *p*-values. The Kruskal–Wallis H test was used to analyze continuous variables. TRG was defined as a binary outcome measure and analyzed using logistic regression models. Multiple logistic regression was performed to identify potentially significant associations, using clinically relevant variables in simple logistic regression. Variable selection was performed using stepwise analysis. Odds ratios (ORs) and 95% confidence intervals (95% CIs) were calculated to evaluate the predictors of TRG. Overall survival (OS) was calculated from surgery to death from any cause or the last date of follow-up. Progression-free survival (PFS) was calculated from surgery to the first event (local recurrence or progression, distant recurrence or death from any cause). Kaplan–Meier estimates of survival rates were compared by log rank tests, and a Cox proportional hazards model was applied to calculate to calculate hazard ratios (HRs), aiming to assess relevant prognostic factors: age, sex, tumor location, Laurén’s classification, use of neoadjuvant ChT, pathologic TNM stage, and molecular subtype. Variable selection for the multivariate Cox model was performed with stepwise analysis. Statistical analyses were performed using SPSS, Version 26 (IBM Corp., Armonk, NY, USA) and RStudio. *p*-value <0.05 was considered statistically significant using 2-sided Cox proportional hazards regression. 

## 3. Results

### 3.1. MSI and EBV Frequency in GC

We included 137 patients with GC; MSI and EBV status was evaluated in all cases on surgical specimens. Clinical and pathologic characteristics of the patients are summarized in Table 1. The series includes 80 (58.4%) male and 57 (41.6%) female patients with a median age at diagnosis of 68 (range 24–90) years. Most tumors were located in the antrum (68 (49.6%)) and were classified as intestinal type according to Laurén’s classification (68 (49.6%)). Ten patients (7.3%) had distant metastases. Fifty-one patients (37.2%) of the whole series had been submitted to POPChT. The majority of the patients (122 (89.1%)) had an R0 resection. Median follow-up was 41 months (IQR 14.5–69 months). At the time of the database lock (30 November 2021), 86 (62.8%) patients had died. 

Concerning MSI status, 96/137 (70.1%) were MSS, 4/137 (2.9%) were MSI-low, and 37/137 (27.0%) were MSI-high. MSI-low cases were grouped with MSS cases for further analysis. Tumors displayed EBV positivity (EBV+) in 7/137 cases (5.1%). 

### 3.2. Clinical and Pathological Characterization 

Tumors were divided into three groups and analyzed according to their molecular subtype: MSS, MSI-high, and EBV+. No cases showed both MSI-high status and EBV positivity. Clinicopathological characteristics of these three groups are shown in Table 1. Patients with MSI-high tumors were more frequently females (59.5% versus 36.6% in MSS/EBV− cases, *p* < 0.001) and had older age (median age of 73 versus 67 years in MSS/EBV−, *p* < 0.001). Regarding histological characterization, more than half of MSI-high tumors were intestinal-type according to Laurén (54.1%) and tubulo-papillary according to WHO (67.6%) classifications. Six out of seven patients (85.7%) with EBV+ tumors were males, and most EBV+ tumors were associated with indeterminate histological subtype (Laurén classification) (*p* < 0.001) or gastric carcinomas with lymphoid stroma, according to the WHO classification (*p* < 0.001). 

### 3.3. Tumor Regression after Neoadjuvant Chemotherapy 

Neoadjuvant ChT regimens are detailed in Table S1. Fifty-one patients were treated with POPChT: in one patient, the type of ChT performed was not available in the medical records; fifty patients received a fluoropyrimidine and platinum-based chemotherapy. Fifteen out of these fifty patients (30.0%) received a triplet regimen including docetaxel (DCF or FLOT), while the remaining thirty-five out of fifty patients (70.0%) received an ECF-like regimen. Forty patients out offifty-one (78.4%) completed all cycles of neoadjuvant ChT. Thirty-four out offifty-one (66.7%) patients had lymph node metastasis on the surgical specimen with a median of four positive lymph nodes (IQR 1.5–9); forty-seven out of fifty-one (92.2%) of these patients had curative resections (R0). Pathological staging and R status in patients submitted to surgery alone or neoadjuvant ChT are shown in Table S2. No differences were found between groups.

TRG in response to neoadjuvant ChT was evaluated in all patients submitted to neoadjuvant ChT according to Becker’s grading system and is represented in Table 2. In this study, none of the 51 patients submitted to neoadjuvant chemotherapy had complete tumor regression, 14 patients had TRG 1b, 16 patients had TRG 2, and 21 patients had TRG3, with more than 50% vital tumor cells. The residual cancers were classified according to Laurén and WHO classifications in keeping with Becker et al. [40]. Morphological and cellular changes related to ChT were also observed, such as necrosis, inflammation, granulation tissue, acellular mucin, fibrosis, cytological atypia, cytoplasmic vacuolization, and multinucleated cells [40]. TRG 1b in the resected specimen was documented in 12/36 (33.3%) of MSS/EBV− tumors and in 2/11 (18.2%) of MSI-high tumors (*p* = 0.500). None of the EBV+ tumors had TRG 1b. 

Due to the low frequency of EBV+ tumors, further analyses seeking predictive factors associated with tumor regression after neoadjuvant ChT were performed considering only MSI-high and MSS/EBV− cases (*n* = 47).

Response to neoadjuvant ChT was analyzed using simple and multiple logistic regression analysis. Regarding ChT regimen, we observed that patients treated without taxanes showed a tendency for worse response on simple logistic regression (OR 1.984, IC 95% 0.48–7.93, *p* = 0.330), but this association was not statistically significant and was not included in the multiple logistic regression model. In the multivariate model, after adjusting for MSI status, the odds of a good pathological response (TRG 1b) were 4.1 times higher in females when compared with male patients (*p* = 0.062). Moreover, the odds of poor pathological regression (TRG 2 and 3) were approximately 4.420 times higher in patients harboring MSI-high GC when compared with MSS/EBV−. Although relevant for the model, this association was not statistically significant. Results concerning simple and multiple logistic regression models are presented in Table 3.

### 3.4. MSI and EBV Subtypes and Survival 

**Figure 1:**
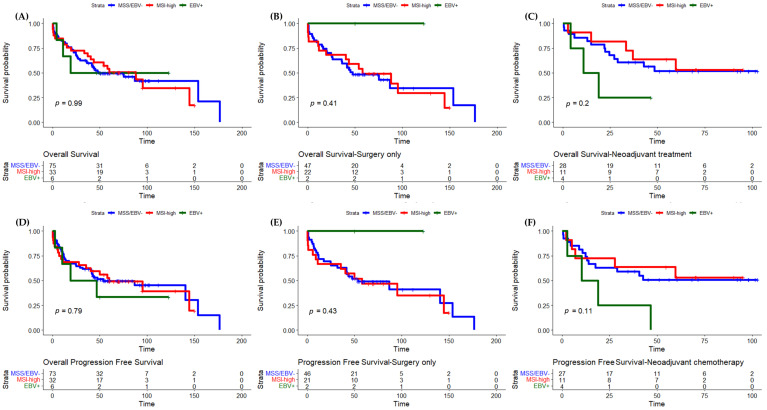
Differences in overall survival and progression-free survival between MSS/EBV−, MSI-high, and EBV+ were assessed using the Kaplan–Meier method and compared using the logrank test. (**A**) OS of the whole cohort. (**B**) OS of patients treated with surgery only. (**C**) OS of patients submitted to neoadjuvant ChT. (**D**) PFS of the whole cohort. (**E**) PFS of patients treated with direct surgery. (**F**) PFS of patients submitted to neoadjuvant ChT. EBV, Epstein–Barr-virus-positive; MSI-high, high microsatellite instability; MSS, microsatellite stable; ChT, chemotherapy.

Patients with metastatic tumors at diagnosis or non-curative resections (R1/R2) were excluded from the survival analysis. The median OS for the whole cohort was 59 months (95% IC 27.523–90.477). No statistically significant differences of OS rates were found between MMS/EBV− and MSI-high (HR 1.004, 95% CI 0.579–1.741, *p* = 0.989) or MMS/EBV− and EBV+ (HR 1.088 95% CI 0.336–3.524, *p* = 0.889) patients (overall log rank *p* = 0.991) (Figure 1A). PFS was also not different between the three groups (log rank *p* = 0.792) (Figure 1D). 

In patients treated with surgery alone, no statistically significant differences regarding the three groups were observed (overall log rank *p* = 0.410), namely MSS/EBV− versus MSI-high (HR 1.004, 95% CI 0.520–1.936, *p* = 0.991) and MSS/EBV−versus EBV+ (HR 0.211, 95% CI 0.006–7.354, *p* = 0.390) (Figure 1B). The two patients with EBV+ tumors had no disease progression and were still alive at the end of follow-up. PFS was longer for patients with MSI-high tumors compared to MSS/EBV− tumors (median 57 months 95% IC 3.0129–110.981 versus median 53 months 95% IC 10.298–95.702), although the difference was not statistically significant (HR 1.130, 95% IC 0.576–2.219, *p* = 0.722) (Figure 1E). 

In patients submitted to neoadjuvant ChT, the survival distributions for the three groups were not statistically significantly different (overall log rank *p* = 0.199): MSS/EBV− versus MSI-high (HR 0.929, 95% CI 0.330–2.614, *p* = 0.890) and MSS/EBV−versus EBV+ (HR 1.465, 95% CI 0.779–2.754, *p* = 0.236) (Figure 1C,F). The same was observed for PFS.

In a multivariate Cox proportional hazards model, only age and (y)pN were included to adjust for the effects of covariates. In this model, age (HR 1.02, IC 95% 1.002–1.056, *p* = 0.033) and positive lymph nodes (HR 1.82; IC 95% 1.034–3.211, *p* = 0.038) were independent prognostic factors for OS (Table 4). 

**Figure 2:**
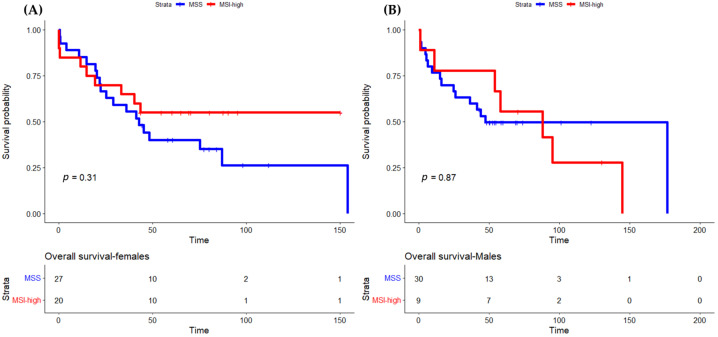
Overall survival by MSI status in females (**A**) and males (**B**). MSI, microsatellite instability; MSS, microsatellite stable.

**Figure 3:**
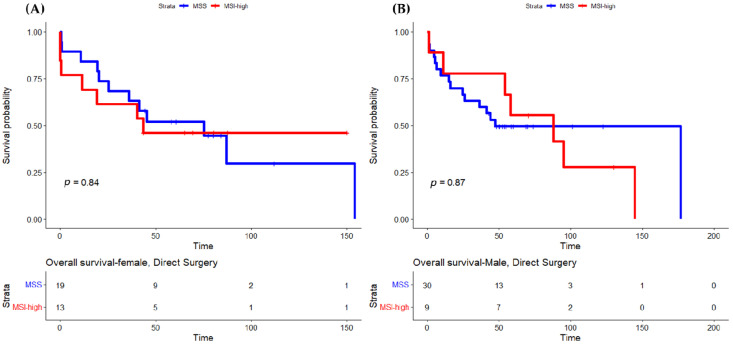
Overall survival by MSI status in females (**A**) and males (**B**) treated with direct surgery. MSI, microsatellite instability; MSS, microsatellite stable.

**Figure 4:**
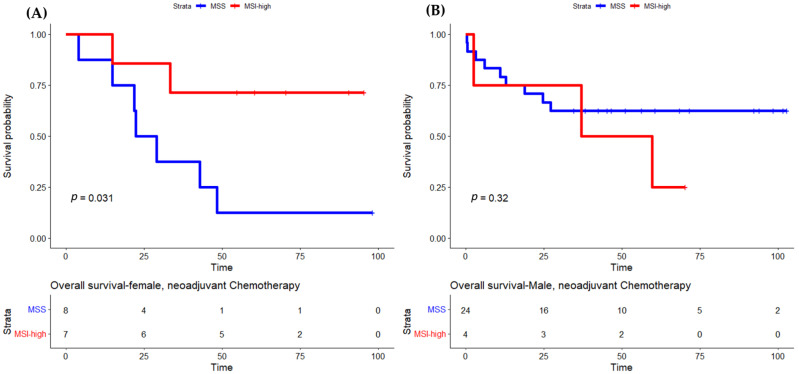
Overall survival by MSI status in females (**A**) and males (**B**) submitted to neoadjuvant chemotherapy. MSI, microsatellite instability; MSS, microsatellite stable.

Concerning sex differences according to MSI status, the OS was not statistically different between female and male patients with MSI-high tumors (HR 0.667, IC 95% 0.269–1.650, *p* = 0.380). When we analyzed females and males with MSS and MSI-high tumors separately, we observed that females with MSI-high tumors had a longer OS compared to females with MSS tumors (HR 0.683, 95% IC 0.306–1.523, *p* = 0.351) (Figure 2). There were no differences in overall survival in patients submitted to surgery (Figure 3). The differences in OS were only statistically significant for female patients treated with POPChT (*p* = 0.031) (Figure 4A). In contrast, the MSI-high subtype seems to be a negative prognostic factor in male patients (Figure 4B). 

Figure S1 shows OS for male and female patients with MSS and MSI-high tumors treated with direct surgery or neoadjuvant ChT. Female patients with MSI-high derived the most benefit from POPChT (HR 0.208, 95% IC 0.042–1.018, *p* = 0.053), followed by males with MSS tumors (HR 0.397, 95% IC 0.147–1.070, *p* = 0.068) and males with MSI-high tumors (HR 0.598, 95% IC 0.149–2.396, *p* = 0.468) when compared to female patients with MSS tumors who showed the worse OS when treated with POPChT.

## 4. Discussion 

In the present study of 137 patients with GC treated at two Portuguese centers either with upfront surgery or with POPChT, we observed an unusually high proportion of MSI-high tumors (27%). This might be related to epidemiologic differences, but it could also be explained by methodological issues as we used the PCR-base molecular analysis, which is the gold standard to assess MSI status [14,22,35,44]. This considerably high proportion of MSI-high tumors contributes to strengthening our findings in this particular molecular subtype of GC.

Overall, clinicopathological characteristics of MSI-high and EBV+ tumors were in line with previous studies [5,45,46,47,48]. MSI-high tumors were more prevalent in older females and more frequently observed in intestinal and tubulo/papillary histologic subtypes according to Laurén and WHO classifications, respectively. EBV+ tumors were observed in 5.1% of patients, and most (86%) were men, which is concordant with previous studies [5,6,22,27,36]. Moreover, as previously described in the literature, the majority (71.7%) of EBV+ GC displayed features of CG with lymphoid stroma (GCLS), which might influence therapeutic options [28,49,50].

Regarding tumor regression analysis after neoadjuvant ChT, we observed that MSI-high tumors were approximately fourtimes more likely to have a poor response to neoadjuvant ChT, but this difference was not statistically significant. In multivariate analysis, we observed that females were more likely to achieve a good response to neoadjuvant ChT, which is in line with a meta-analysis performed by Athauda et al. [51]. Thus, females with MSI-high tumors did far better than females with MSS/EBV− tumors when treated with POPChT. In contrast, in male patients with MSI-high tumors, a tendency for lower OS was observed, whether they went for direct surgery or were treated with POPChT. Age (HR 1.02, IC 95% 1.002–1.056, *p* = 0.033) and positive lymph nodes (HR 1.82; IC 95% 1.034–3.211, *p* = 0.038) werethe only independent factors to influence OS. Molecular subtypes did not affect OS or PFS either in patients treated with surgery alone or in those submitted to POPChT. 

We think that these results add important information to the current literature in terms of deciding whether patients with locally advanced GC of the MSI-high subtype derive any benefit from POPChT.

In Europe, and since the publication of MAGIC trial, POPChT is the standard of care for patients with locally advanced GC [3,52]. Later on, Al-Batran et al. observed that a regimen containing a taxane (FLOT—fluorouracil, leucovorin, oxaliplatin, and docetaxel) was superior to ECF in terms of OS and relapse-free survival [47]. In our cohort, all patients received regimens with fluoropyrimidine and platin (cisplatin or oxaliplatin), and 15/51 (29.4%) were treated with a regimen including docetaxel (FLOT-like). 

Current treatment approaches in resectable GC are based mainly on clinical TNM staging to decide whether the patient goes for direct surgery or for POPChT. Accordingly, tumors with >IB stage should be treated with POPChT, while in the remaining patients, direct surgery or even endoscopic resection in T1a tumors is recommended [3,53]. The first study to question whether all patients should be treated equally, independently of the molecular subtype of the tumors, was the post hoc analysis of the MAGIC trial where, in an exploratory analysis, the authors found that MSI-high subtype was a negative predictive marker for ChT efficacy based on the lack of histopathological response in all nine patients with MSI-high tumors treated with neoadjuvant ChT [19]. 

Subsequent studies addressing this issue provided discrepant results but somewhat supporting the lack of response to ChT in MSI-high tumors. However, it is worth noting that most of these series include only Asian patients, whose surgical and chemotherapy regimens differ substantially from those of European protocols [23,34,39]. Careful analysis of each of these studies is mandatory before conclusions are drawn on whether specific subtypes respond to POPChT.

The study by Pietrantonio et al. is a meta-analysis including over 1500 patients from four large RCTs: MAGIC, CLASSIC, ARTIST, and ITACA-S. About two thirds of these patients (CLASSIC and ARTIST) were Asian with the above-mentioned limitations [33]. Of the 576 European patients, 317 were those included in the MAGIC trial, and 259 were from the ITACA trial, where patients were randomly assigned to receive two different schedules of adjuvant ChT [52,54]. As such, if no patients were treated with surgery alone, it is not possible to conclude whether MSI-high is a negative predictor to ChT. 

In another study by Choi et al., patients from the CLASSIC trial were analyzed. This Asian trial compares surgery only to adjuvant capecitabine and oxaliplatin. Authors observed that patients with MSI-high tumors did not derive any survival benefit from adjuvant ChT [55]. Later on, Rohet al. focused on this same population but used a single patient classifier (SPC) based on the expression of nine genes, defining prediction of ChT benefit as SPC prediction (responder or non-responder). The effect of MSI and EBV+ on response to ChT was analyzed according to classes of SPC and not by itself [23]. In contrast to these results, a systematic review and meta-analysis including seven studies, mostly Asian, showed that disease-free survival (DFS) and OS were longer for the deficient MMR/MSI-high patients treated with adjuvant ChT than for those treated with surgery alone [56].

In contrast, the study by Kohlrusset al. includes 617 German patients evaluated for MSI and EBV status, of whom 291 were treated with upfront surgery and 326 with POPChT. The latter included platinum/5-FU-based regimens, but a proportion of patients also received taxanes, which also differs from previous series analyzed. In this large group of patients treated in a homogeneous manner, the authors observed that MSI-high status was not predictive of response to neoadjuvant platinum/5-FU-based ChT, with or without taxanes, but it was indicative of a better prognosis. This is one of the few studies that analyzes MSI-low tumors separately [36]. 

In conclusion, if we only focus on European series where patients are treated with neoadjuvant ChT with a 5-FU/cisplatin-based regimen, some of which also received taxanes, there is no evidence that MSI-high tumors respond differently from MSS tumors. An Italian multicentric and observational study in the real-world setting demonstrated that perioperative FLOT is feasible and safe in patients with resectable GC in clinical daily practice. Although pathologic complete response was lower than expected, it was still identified as a predictive marker of survival [57]. MSI-high status was suggested to be a positive prognostic marker also in patients treated with a taxane-containing triplet (FLOT) in the perioperative setting. These findings, reported outside clinical trials, are essential. This is also supported by our study, where we observed a trend for better response in patients treated with taxanes. With respect to MSI-high tumors, we observed that they were more likely to have a poor response to POPChT, but again differences were not statistically significant. As such, we conclude that all these recent European studies are in agreement with the recently published ESMO guidelines regarding peri-operative treatment of MSI-high disease, where the authors emphasize that data from a small number of MSI-high patients treated with FLOT demonstrated better response rates than historical rates with platinum–5-FU [58]. 

Data in neoadjuvant ChT in EBV+ resectable GCs are even more limited. Kohlruss et al. found better OS for seven EBV+ GCs after primary resection compared to five treated with neoadjuvant ChT, while Biesma et al. found that EBV+ tumors showed the highest histopathological response rate compared to MSS/EBV− [36,37]. In our series, none of the patients with EBV+ tumors responded to POPChT, and they seemed to do better with direct surgery, but differences are not statistically significant. 

Regarding the better prognosis of MSI-high cases reported by most studies, in the present study, we found that this prognostic effect was highly dependent on sex. Females with MSI-high tumors had longer OS than those with MSS GC when treated with POPChT. The opposite was observed in males: those with MSI-high tumors had a lower OS and did worse when treated with POPChT compared to males with MSS tumors, but differences were not statistically significant. These findings are in line with previous studies addressing the impact of sex on survival in patients with GC treated or not with POPChT. Quaas et al. showed that the favorable prognostic value of MSI was only seen in females and not in males [37]. In a meta-analysis including four randomized controlled trials of patients with localized esophagogastric cancers submitted to ChT, females had improved OS compared to males and were more likely to achieve favorable histologic tumoral regression after ChT (*p* = 0.10) [51]. Finally, a study by Kolhruss et al. showed a superior survival of women with MSI-high gastric and gastroesophageal tumors after neoadjuvant ChT, with a considerable interaction between sex and MSI in this group of patients [38]. 

Data from different parts of the world have shown that men are both at increased risk and have higher mortality compared with women for most cancers [59,60].However, little is known about sex-specific differences regarding OS and response to neoadjuvant ChT in the setting of GC. Noteworthy is the fact that women are often under-represented in clinical trials, which is a potential limitation to demonstrating significant interactions between sex and treatment. In our study, we found a higher frequency of females (41.6%) compared to previous series [38,51,61]. 

Several factors may explain sex disparity in terms of survival, including behavioral and biologic aspects, sex-biased gene expression signatures or tumor-specific molecular changes, different immune responses, and sex influence in pharmacokinetics and pharmacodynamics [38,51,60,62,63]. In general, women show stronger innate and adaptative immune reactions than men, being considered “immune hot”, which can increase response to chemotherapy and might lower their cancer mortality. Sex-biased genetic alterations might influence response to different treatments. For example, Grosser et al. observed that p53 mutations were associated with the male sex and patients with mutated p53, and MSI-high tumor showed a worse survival when treated with neoadjuvant ChT [64]. 

Finally, MSI-high has been identified as a biomarker for response to immunotherapy, but the effect of sex needs to be explored [4]. Confortiet al. carried out a meta-analysis showing that immune checkpoint inhibitors (ICIs) are more effective in men than women, while women obtain more clinical benefit from ICIs combined with ChT [61,65]. In metastatic gastric/gastroesophageal adenocarcinoma, ICIs compared to standard treatment had a non-significantly greater effect in males and only increased survival in the MSI-high subgroup [66]. Ge et al. explored sex variance in GC somatic mutation profiles and observed that ATRX (tumor gene suppressor involved in DNA damage repair) mutations occurred more frequently in female GC and correlated to the MSI-high subtype, better overall survival, and favorable clinical benefit to ICI in patients with GC [62].

Altogether, these findings suggest that therapeutic response and prognosis are sex-modulated and dependent on molecular subtype. Information on gender is readily available and should probably be taken into consideration for selecting patients for chemotherapy.

Our study has some limitations. First, due to its retrospective nature, it should be considered an exploratory analysis, as it includes data collected from clinical charts certainly with some imprecisions regarding patient related factors and treatment protocols. The number of patients with MSI-low and EBV+ tumors is also small, which precludes definitive conclusions regarding these molecular subtypes. As points of strength, our series consists of a homogeneous group of patients treated in the same manner, with a substantial proportion of them performing POPChT (37%), both with and without taxanes. Finally, pathologic examination of the surgical specimen, namely molecular subtype and TRG, was always reviewed centrally by two dedicated GI pathologists.

## 5. Conclusions

In our study, we found a high frequency of MSI-high tumors compared to most published series. The present study adds further evidence to the recently published ESMO 2022 guidelines that MSI-high GCs should not be excluded from POPChT with the FLOT regimen [3,20,36]. Regarding tumor regression status, females had a better chance of responding to POPChT. The better prognosis observed in MSI-high cases was only observed for female patients, whereas in males, a trend for a worse prognosis was observed. These are important findings that, if confirmed by other studies, may help to pave the way to tailored treatment in GC considering both patients’ and disease characteristics, besides clinical TNM staging as of today.

## Figures and Tables

**Table 1:** Clinical and morphologic characterization for all tumors and according to molecular subtype.

Variables	All	MSS/EBV−	MSI-High	EBV+	*p*-Value ^a^
Total, *n* (%)	137 (100)	93 (67.9)	37 (27.0)	7 (5.1)	
Sex Male Female	80 (58.4) 57 (41.6)	59 (63.4) 34 (36.6)	15 (40.5) * 22 (59.5) *	6 (85.7) 1 (14.3)	<0.013
Age at diagnosis(years) median (range)	68 (24–90)	67 (24–87)	73 (41–90) *	60 (44–82)	<0.001
Tumor location Fundus/Proximal Body/Middle Antrum/Distal	33 (24.1) 36 (26.3) 68 (49.6)	27 (29.0) 23 (24.7) 43 (46.2)	3 (8.1) 12 (32.4) 22 (59.5)	3 (42.9) 1 (14.3) 3 (42.9)	0.108
Laurén classification Intestinal Diffuse Mixed Indeterminate	68 (49.6) 29 (21.1) 16 (11.7) 24 (17.5)	47 (50.5) 23 (24.7) 14 (15.1) 9 (9.7) *	20 (54.1) 6 (16.2) 1 (2.7) 10 (27.0)	1 (14.3) 0 (0.0) 1 (14.3) 5 (71.4) *	<0.001
WHO classification Tubulo/papillary Poorly cohesive Mucinous Mixed GCLS Adenosquamous ^c^	77 (56.2) 29 (21.2) 2 (1.5) 16 (11.7) 12 (8.8) 1 (0.7)	51 (54.8) 23 (24.7) 2 (2.2) 14 (15.1) 3 (3.2) * 0 (0.0)	25 (67.6) 6 (16.2) 0 (0.0) 1 (2.7) 4 (10.8) 1 (2.7)	1 (14.3) 0 (0.0) 0 (0.0) 1 (14.3) 5 (71.4) * 0 (0.0)	<0.001
Pathologic (y)T ^b^ T1/2 T3/4	47 (34.3) 90 (67.7)	32 (34.4) 61 (65.6)	12 (32.4) 25 (67.6)	3 (42.9) 4 (57.1)	0.888
Pathologic (y)N ^b^ Negative Positive	45 (32.8) 92 (67.2)	31 (33.3) 62 (66.7)	13 (35.1) 24 (64.9)	1 (14.3) 6 (85.7)	0.530
Resection status R0 R1 R2	122 (89.1) 9 (6.6) 6 (4.3)	80 (86.0) 7 (7.5) 6 (6.5)	35 (94.6) 2 (5.4) 0 (0.0)	7 (100) 0 (0.0) 0 (0.0)	0.445
Neoadjuvant ChT No Yes	86 (62.8) 51 (37.2)	57 (61.3) 36 (38.7)	26 (70.3) 11 (29.7)	3 (42.9) 4 (57.1)	0.377

EBV, Epstein–Barr virus; ChT: chemotherapy; GCSL, gastric carcinoma with lymphoid stroma; MSI-high, high microsatellite instability; MSS: microsatellite stable; ^a^ *p*-value of chi-squared test or Fisher’s exact test. The Kruskal–Wallis H test was used to analyze the continuous variable “age”; ^b^ classification according to eighth edition AJCC system for GC. y denotes the T, N, and TNM stages after neoadjuvant chemotherapy; ^c^ due to its low representation, adenosquamous tumor was not included in the comparison analysis; * significant *p*-value after Bonferroni correction, results from chi-squared post hoc test for multiple comparisons.

**Table 2:** Tumor regression status after neoadjuvant chemotherapy.

Tumor Regression Grade ^a^	MSS/EBV− *n* = 36	MSI-High *n* = 11	*p*-Value ^b^	EBV+ *n* = 4	*p*-Value ^b^
TRG 1b (<10% residual tumor)	12 (33.3)	2 (18.2)	0.500	0 (0.0)	0.380
TRG 2 (10–50% residual tumor)	11 (30.6)	3 (27.3)		2 (50.0)	
TRG 3 (>50% residual tumor)	13 (36.1)	6 (54.5)		2 (50.0)	

EBV, Epstein–Barr virus; MSI-high, high microsatellite instability; MSS, microsatellite stable; ^a^ TRG according to Becker classification; ^b^ *p*-value of chi-squared test or Fisher’s exact test compared to MSS/EBV−.

**Table 3:** Univariate andmultivariate analysis of tumor regression status after neoadjuvant chemotherapy.

	TRG		Simple Logistic Regression ^a^			Multiple Logistic Regression ^b^		
Variables	TRG 1b *n* = 14	TRG 2 + 3 *n* = 33	OR	95% CI	*p*-Value	OR	95% CI	*p*-Value
Sex								
Female	7	9	1.00			1		0.062
Male	7	24	2.66	0.72–10.06	0.138	4.10	0.97–19.95	
Age	64.5	65.0	0.995	0.940–1.0489	0.864	Excluded		
Type of neoadjuvant ChT								
Taxanes	5	7	1.00					
Without taxanes	9	25	1.984	0.48–7.93	0.330	Excluded		
Laurén Classification								
Intestinal	8	19	1.00					
Non-intestinal	6	14	0.982	0.27–3.59	0.97	Excluded		
WHO classification								
Tubulo/papillary	8	23	1.00					
Poorly cohesive	2	7	1.217	0.231–9.308	0.827	Excluded		
Mucinous	0	1	5.443952 × 10^6^	3.083917 × 10^−206^–NA *	0.994			
Mixed	2	1	0.173	0.0074 2.049	0.175			
GCLS	2	1	0.173	0.0074–2.049	0.175			
MSI status								
MSS/EBV−	12	24	1.00			1.00		0.124
MSI-high	2	9	2.25	0.48–16.30	0.344	4.420	0.78–38.96	

EBV, Epstein–Barr virus; ChT, chemotherapy; GCSL, gastric carcinoma with lymphoid stroma; MSI-high, high microsatellite instability; MSS, microsatellite stable; NA, non-applicable; TRG, tumor regression grade; ^a^ univariate logistic regression was analyzed considering all patients submitted to ChT (*n* = 47); ^b^ in multiple logistic regression, one patient was excluded because the type of ChT performed was not available in the medical records; * Since there were no patients with TRG1b mucinous tumors, a high unreliable OR was obtained and upper limit of 95% IC was not possible to compute.

**Table 4:** Simple and multiple Cox proportionalhazards model for analysis of the OS of patients with operated localized GC.

	Simple Cox Model					Multiple Proportional Hazards Cox Model				
Variables	Coef	SE	HR	IC 95%	*p*-Value	Coef	SE	HR	IC 95%	*p*-Value
Sex										
Female			1.00			Excluded				
Male	−0.153	0.255	0.858	0.519–1.416	0.549					
Age	0.0225	0.013	1.026	0.994–1.054	0.055	0.028	0.013	1.02	1.002–1.056	0.033
Tumor location						Excluded				
Fundus/Proximal			1.00							
Body/Middle	−0.270	0.344	0.762	0.388–1.500	0.432					
Antrum/Distal	−0.305	0.311	0.736	0.4003–1.355	0.325					
Laurén classification						Excluded				
Intestinal			1.00							
Non-intestinal	−0.1281	0.258	0.887	0.53–1.46	0.62					
Neoadjuvant ChT						Excluded				
No			1.00							
Yes	−0.107	0.270	0.898	0.528–1.528	0.692					
(y)pT ^a^						Excluded				
T1/T2			1.00							
T3/T4	0.296	0.270	1.345	0.791–2.287	0.273					
(y)pN ^a^										
Negative			1.00							
Positive	0.539	0.287	1.714	0.976–3.01	0.060	0.599	0.289	1.82	1.034–3.211	0.038
Molecular classification						Excluded				
MSS/EBV−			1.00							
MSI-high	0.004	0.280	1.00	0.579–1.741	0.989					
EBV (+)	0.084	0.599	1.087	0.336–3.524	0.889					

EBV, Epstein–Barr virus; ChT, chemotherapy; MSI-high, high microsatellite instability; MSS, microsatellite stable. ^a^ Classification according to eighth edition AJCC system for GC. y denotes the T, N, and TNM stages after neoadjuvant chemotherapy.

## Data Availability

The data presented in this study are available in this article and the Supplementary Materials.

## Supplementary Materials

The following supporting information can be downloaded at: https://www.mdpi.com/article/10.3390/cancers15010074/s1, Table S1: Neoadjuvant chemotherapy regimens according to molecular classification; Table S2: Clinical characterization and pathological staging for all tumors according to treatment type (surgery alone versus neoadjuvant ChT); Figure S1: Overall survival by MSI status in females and males treated with direct surgery (A) or perioperative chemotherapy (B).
