# Supplementary material for: EBV and MSI Status in Gastric Cancer: Does It Matter?
Source: Cancers (Basel). 2022 Dec 22;15(1):74. doi: 10.3390/cancers15010074 (PMC9817503; doi:10.3390/cancers15010074)
Supplement: Supplementary file 1 [file cancers-15-00074-s001.zip › cancers-2085368-supplementary.pdf]

Supplementary data

EBV and MSI Status in Gastric Cancer: Does It Matter?

Catarina Neto do Nascimento, Luís Mascarenhas-Lemos, João Ricardo Silva, Diogo Sousa Marques, Catarina Ferreira Gouveia, Ana Faria, Sónia Velho, Rita Garrido, Rui Maio, Andreia Costa, Patrícia Pontes, Xiaogang Wen, Irene Gullo, Marília Cravo, Fátima Carneiro

Table S1. Neoadjuvant chemotherapy regimens according to molecular classification.

Regimen – n (%)	All tumors (n=50)*	MSS/EBV- (n=35)*	MSI-high (n=11)	EBV+ (n=4)
ECF-like	34 (66.7)	24 (68.6)	9 (81.9)	1 (25.0)
FLOT	7 (13.7)	6 (17.1)	1 (9.1)	0 (0.0)
FOLFOX	1 (2.0)	1 (2.9)	0 (0.0)	0 (0.0)
DCF-like	8 (15.7)	4 (11.4)	1 (9.1)	3 (75.0)

ECF: epirubicin + cisplatin +fluorouracil; FLOT: fluorouracil +leucovorin+ oxaliplatin+docetaxel; FOLFOX: leucovorin + oxaliplatin + fluorouracil ; DCF: docetaxel +cisplatin + fluorouracil; *in one patient with MSS tumor the type of ChT performed was not available in the medical records (n=50).

Table S2. Clinical characterization and pathological staging for all tumors according to treatment type (surgery alone *versus* neoadjuvant ChT).

Variables	Surgery alone	Neoadjuvant ChT	p-value[a]
**Total – n (%)**	**86 (62.8)**	**51 (37.2)**	
**Sex**			
Male	46 (53.5)	34 (66.7)	0.130
Female	40 (46.5)	17 (33.3)	
**Age at diagnosis** (years) median (range)	70 (41-90)	64 (24-81)	0.001
**Pathologic (y)T[b]**			
T1/2	32 (37.2)	15 (29.4)	0.353
T3/4	54 (62.8)	36 (70.6)	
**Pathologic (y)N**			
Negative	28 (32.6)	17 (33.3)	0.926
Positive	58 (67.4)	34 (66.7)	
**Resection status**			
R0	75 (87.2)	47 (92.2)	0.608
R1	7 (8.1)	2 (3.9)	
R2	4 (4.7)	2 (3.9)	
**MSI/EBV status**			
MSS/EBV-	58 (67.4)	36 (70.6)	0.383
MSI-high	54 (29.1)	11 (21.6)	
EBV+	3 (3.5)	4 (7.8)	

EBV, Epstein-Barr virus; ChT, chemotherapy; MSI-high, high microsatellite instability; MSS, microsatellite stable; [a]p-value of Chi-square test or Fisher's exact test compared to MSS/EBV−. The Kruskal-Wallis H test was used to analyze the continuous variable "age".[b] Classification according to 8th edition AJCC system for GC. y denotes the T, N, and TNM stages after neoadjuvant chemotherapy.

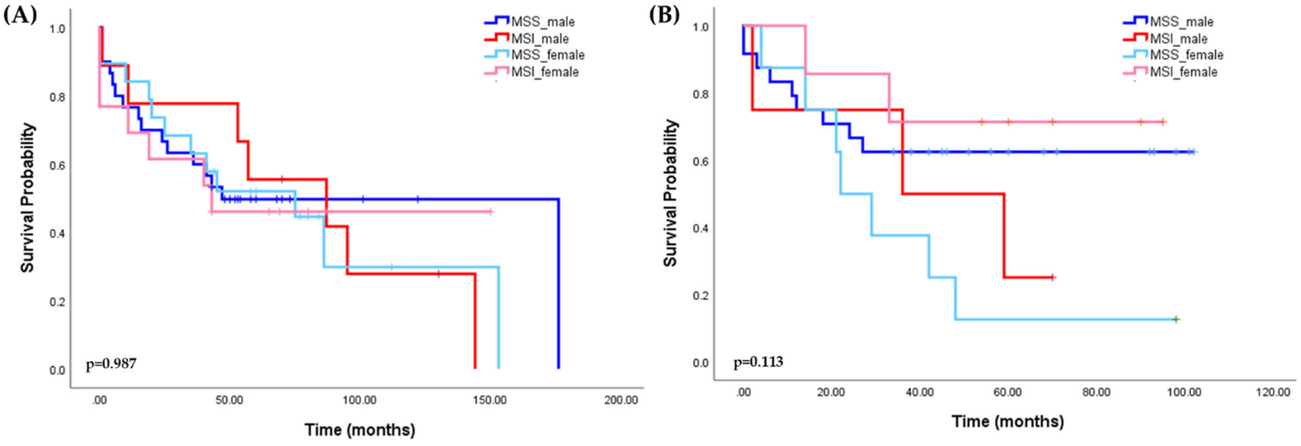

Figure S1. Overall survival by MSI status in females and males treated with direct surgery (A) or perioperative chemotherapy (B). MSI microsatellite instability; MSS, microsatellite stable.
